# Enhanced Thermoelectric Performance of Indacenodithiophene-Benzothiadiazole Copolymer Containing Polar Side Chains and Single Wall Carbon Nanotubes Composites

**DOI:** 10.3390/polym12040848

**Published:** 2020-04-07

**Authors:** Zhongming Chen, Tongchao Liu, Chengjun Pan, Guiping Tan

**Affiliations:** 1 School of Environment and Civil Engineering, Dongguan Cleaner Production Technology Center, Dongguan University of Technology, Dongguan 523808, China; liu21645@163.com; 2 Shenzhen Key Laboratory of Polymer Science and Technology, College of Materials Science and Engineering, Shenzhen University, Shenzhen 518060, China; pancj@szu.edu.cn

**Keywords:** organic thermoelectric materials, interfacial interactions, composites

## Abstract

Composite films of indacenodithiophene-bezothiadazole copolymers bearing polar side chains (**P1**) and single wall carbon nanotubes (SWCNTs) are found to show a competitive thermoelectric performance compared to their analogous polymers with aliphatic side chains (**P2**). The enhanced power factors could be attributed to the stronger interfacial interactions between the **P1**/SWCNTs compared to that of **P2**/SWCNTs containing the same ratio of SWCNTs. A maximum power factor of 161.34 μW m^−1^ K^−2^ was obtained for the composite films of **P1**/SWCNTs for a filler content of 50 wt%, which is higher than that of **P2**/SWCNTs (139.06 μW m^−1^ K^−2^, 50 wt%). Our work sheds light on the design of side-chains in efficient conjugated polymers/SWCNTs thermoelectric materials and contributes to the understanding of their thermoelectric properties.

## 1. Introduction

In the development of next-generation green energy materials, great attention has been paid to organic thermoelectric materials (OTEs) which can directly convert thermal energy into electricity [1,2,3,4,5,6,7]. Conjugated polymers (CPs) are an intriguing class of semiconductors widely used in the field of organic electronics (such as organic light-emitting diodes (OLED), organic field-effect transistors (OFETs), sensors, etc.) [8,9,10,11,12] due to their low toxicity, low thermal conductivity and mechanical flexibility. Recent results have shown that CPs are among the most promising materials for application in flexible thermoelectric generators [13,14,15,16,17]. For example, poly(3,4-ethylenedioxy- thiophene) (PEDOT) with a high thermoelectric figure of merit (*ZT*) of 0.45 has been reported [18], which stimulated the investigation of CPs to be used for thermoelectric generators.

In order to achieve high performance thermoelectric materials, both large a Seebeck coefficient (S) and high electrical conductivity (σ) are necessary according to the following equation [19]:

*ZT* = S^2^σT/κ (1)

Organic materials generally possess large Seebeck coefficients (S) and low thermal conductivity [13,14,15,16,17,18,19], therefore, the main challenge of achieving high-performance OTEs is how to improve the electrical conductivity of CPs without sacrificing their large Seebeck coefficients and low thermal conductivity [20,21]. Many strategies have been attempted to improve the conductivities of CPs (such as structural manipulations [22], strengthening the ordering of polymer packing [23], efficient doping [24], and forming composites with highly conductive inorganic materials [25], etc.).

CPs/inorganic thermoelectric composites are one of the major types of organic thermoelectric materials which combine the large Seebeck coefficient feature of CPs with the low thermal conductivity and high electrical conductivities of inorganic nanomaterials [26,27,28,29,30,31,32,33]. Single wall carbon nanotubes (SWCNTs) are one type of attractive carbon nanomaterial for forming thermoelectric composites, attributed to their high electric conductivity and excellent flexibility [27]. Therefore, various kinds of CPs/SWCNTs composites have been prepared and their thermoelectric properties were investigated [26,27,28,29,30,31,32,33]. It was found that the interfacial interactions between SWCNTs and CPs are crucial to enhance the charge carrier mobility for the composites. Thus a few attempts to fine tune the interfacial interactions of the composites and improve their thermoelectric performance through enhancing the charge carrier mobility have been reported [34,35,36].

The introduction of polar side-chains into CPs has been considered an efficient method to increase the interfacial interactions between CPs and SWCNTs, thus improving the thermoelectric performance. For example, Bazan et al. found that conjugated polyelectrolytes (CPEs) with pendant ionic functionalities can serve as good dispersants and efficient dopants of SWCNTs, allowing a series of p-type or n-type solution-processable composites to be prepared [37]. Crispin et al. demonstrated that optimally p-doped poly(3-hexylthiophene) (P3HT)/CNT composite films could raise the thermoelectric properties to a competitive level (maximum power factor: 95 ± 12 μW m^−1^ K^−2^) [38].

Herein, we report the synthesis of two indacenodithiophene (IDT)-benzothiadiazole-based copolymers bearing polar side chains (**P1**) and aliphatic side chains (**P2**), respectively. Polymer/ SWCNTs composites with different amounts of SWCNTs have been prepared. The effect of the polar side chain on the properties of the intrinsic polymers as well as the composites have been thoroughly investigated. We found that the polar polymer side chains have no effect on the photophysical and electronic features of the polymer backbone, however, their interfacial interactions are stronger than those of polymers with aliphatic side chains. The enhanced interfacial interactions of **P1**/SWCNTs provide the composites with a power factor of 161.34 μW m^−1^ K^−2^ (50 wt%), larger than that of **P2**/SWCNTs (139.06 μW m^−1^ K^−2^) for the same compositing ratio.

## 2. Materials and Methods

### 2.1. General Conditions

^1^H-NMR spectra of the polymers were acquired on an AVANCE III 600 MHz NMR spectrometer (Bruker, Fällanden, Switzerland) at room temperature in CDCl_3_. Chemical shifts are expressed in parts per million (ppm, *δ* scale) and are referenced to tetramethylsilane (TMS). Thermal gravimetric analysis (TGA) was performed on a TGA-55 instrument (TA Instruments, NewCastle, DE, USA) from room temperature to 700 °C under a 20 mL min^−1^ nitrogen flow and a heating rate of 10 °C min^−1^. The molecular weights and polydispersity index (PDI) of the polymers were determined by gel permeation chromatography (GPC) (Waters e2695 Separations Module, Waters, Singapore) using THF as the eluent in the room temperature, and polystyrene was used as a standard. Differential scanning calorimetry (DSC) was performed on a DSC7020 instrument (DSC7020, Tokyo, Japan), in which three polymers were measured in a temperature range from 0 °C to 300 °C at a heating rate of 10 °C min^−1^, opened pans without caps were used in the DSC experiments. Ultraviolet–visible–near-infrared (UV-vis-NIR) absorption spectra were acquired using a Lambda 950 spectrophotometer (PerkinElmer, Waltham, MA, USA), using thin films samples casted on quartz plates. The morphology of the polymer films was observed by a SU-70 scanning electron microscope (SEM) (Hitachi SU-70, Tokyo, Japan), and the thickness of the films was obtained on a Surfcorder ET 4000M (Kosaka Laboratory, Tokyo, Japan). The Raman spectra were recorded on a Renishaw in Via-Reflex Raman microscope (Renishaw inVia™ Raman Microscope, London, England). Cyclic voltammetry (CV) was performed on a CHI 660E electrochemical workstation (Chenhua Instruments Co., Shanghai, China). A platinum plate, Ag/AgCl and a platinum wire were used as the working, reference and counter electrodes, respectively, in 0.1 M tetrabutylammonium hexafluorophosphate (Bu_4_NPF_6_)/acetonitrile under a nitrogen atmosphere at a scan rate of 50 mV s^−1^. The reference electrode was calibrated with ferrocene/ferrocenium (Fc/Fc^+^). Grazing incidence X-ray diffraction (GI-XRD) was measured on a SmartLab X-ray diffractometer (Rigaku, Tokyo, Japan) with a copper target (*λ* = 1.54 Å), and the incident range was 2–40°, the thin films samples are casted on the quartz plates. The electrical conductivity (σ) and Seebeck coefficient (S) of the polymer films were collected using an MRS-3 thin-film thermoelectric test system (Wuhan Joule Yacht Science & Technology, Wuhan, China). The thin films samples are casted on quartz plates, and the measurements are carried out under vacuum at room temperature.

### 2.2. Syntheses of the Monomers and Copolymers

#### 2.2.1. Synthesis of the Monomer M1

**M1** was synthesized using a previously reported method [39]. A mixture of di(ethylene glycol) ethyl ether (0.300 g, 2.23 mmol) and THF (6 mL) was added to a 50 mL two-necked flask. The flask was evacuated and refilled with nitrogen several times, then sodium hydride (NaH) powder was added slowly at 0 °C, and the reaction mixture was stirred for 3 h. 4,7-Dibromo-5,6-difluoro-2,1,3-benzothiadiazole (0.409 g, 1.24 mmol) dissolved in anhydrous THF (10 mL) was then added via syringe, and the reaction mixture was gradually warmed to room temperature. After stirring for 15 h, the reaction mixture was diluted with dichloromethane (DCM) and washed sequentially with water (3 times) and brine. The organic layer was dried over magnesium sulphate (MgSO_4_) and concentrated under reduced pressure. The crude product was purified through silica gel column chromatography (DCM/hexane: 3/4), to afford a white powder (yield: 50 %). ^1^H-NMR *δ* (ppm): 1.16 (dd, 6H, CH_3_), 3.51-3.43 (m, 4H, CH_2_), 3.59–3.52 (m, 4H, CH_2_), 3.73–3.67 (m, 4H, CH_2_), 3.97–3.90 (m, 4H, CH_2_), 4.48–4.42 (m, 4H, CH_2_) (Figure S1 in Supplementary Materials).

#### 2.2.2. Synthesis of the Polymers P1 and P2

**Scheme 1:**
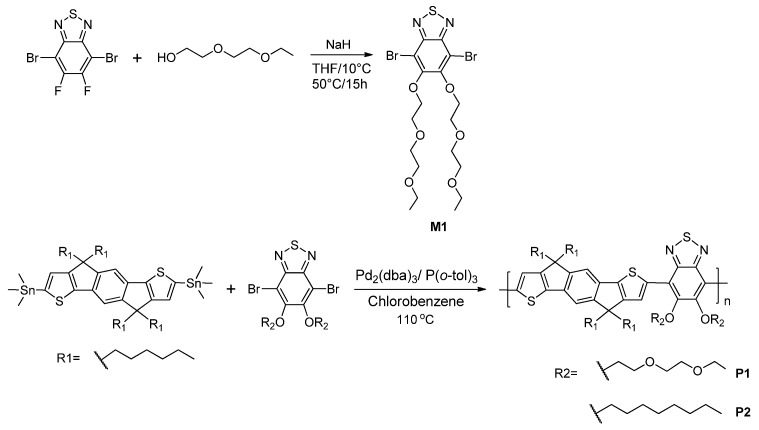
Synthetic routes to the monomer **M1** and copolymers **P1** and **P2**.

**P1** and **P2** were synthesized using a previously reported method [40] (shown in Scheme 1). The detailed procedure is as follows: a mixture of 2,7-dibromo-4,4,9,9-tetraoctyl-4,9-dihydro-*s*-indaceno[1-*b*:5,6-*b*’]-dithiophene (0.200 g, 0.229 mmol), bis(trimethylstannyl) compound (0.229 mmol), Pd_2_(dba)_3_ (0.011 g, 0.011 mmol), and P(*o*-tol)_3_ (0.018 g, 0.057 mmol) in anhydrous chlorobenzene (5 mL) with a nitrogen flow was sealed and stirred for 72 h at 110 °C. After the mixture was cooled to room temperature, the polymer was precipitated by the addition of excess methanol. The precipitate was sequentially washed with methanol, acetone, and deionized water. After drying under vacuum, the polymers were obtained as black powders.

**P1**: black powder, Yield 66 %, ^1^H-NMR *δ* (ppm): 1.35–0.49 (m, 50H, CH_2_–CH_3_), 2.32–1.77 (m, 8H, CH_2_), 4.66–3.23 (m, 20H, CH_2_), 7.44 (s, 2H, aromatic), 8.19 (s, 1H, thiophene), 8.57 (s, 1H, thiophene) (Figure S2).

**P2**: black powder, Yield 56 %, ^1^H-NMR δ (ppm): 1.49–0.66 (m, 74H, CH_2_–CH_3_), 2.23–1.89 (m, 8H, CH_2_), 4.21 (m, 4H, CH_2_), 7.48–7.35 (m, 2H, aromatic), 8.42 (d, 2H, aromatic) (Figure S3).

### 2.3. Preparation of the Thermoelectric Composites

**Scheme 2:**
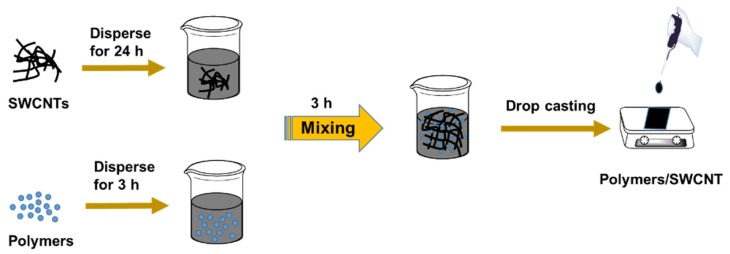
Schematic illustration showing the preparation process for the conjugated polymers (CPs)/single wall carbon nanotubes (SWCNTs) composite films.

The CP/SWCNT composite films were prepared by a drop-casting method following the preparation procedure shown in Scheme 2. Glass slides (10 mm × 10 mm) were sequentially washed with acetone, ethanol, and deionized water in an ultrasonic bath for 30 min. Then SWCNTs (20 mg) were dispersed in anhydrous chlorobenzene (20 mL) to form a 1 mg mL^−1^ suspension; the polymer itself is also prepared as a 1 mg mL^−1^ solution. By mixing different amounts of polymer solutions and SWCNT suspensions we formed CPs/SWCNT composites with various amounts of SWCNTs. In a separate beaker, specific amounts of SWCNTs solution and polymer solutions are taken from the stock solutions by using pipettes and added to the beakers. The obtained mixtures were further sonicated for 3 h to produce homogeneous suspensions. Finally, the suspensions were drop−cast onto the surface of the glass slides to form the composite films after evaporating the solvents.

## 3. Results and Discussion

### 3.1. Molecular Design, Synthesis, and Characterizations of the Copolymers

The monomer **M1** with attached polar oligoethylene glycol side chains was prepared by an in situ alkylation reaction of 2-(2-methoxyethoxy)ethanol with 4,7-dibromo-5,6-difluoro-2,1,3-benzothiadiazole. The polymers **P1** and **P2** were synthesized via Stille coupling in 80% yield by using Pd_2_(dba)_3_ as a catalyst and toluene as a solvent. The detailed synthetic procedures are described in the Materials and Methods section. The chemical structures of the obtained polymers are characterized by ^1^H-NMR spectroscopy. By size exclusion chromatography (SEC) in tetrahydrofuran (THF), the number-average molecular weights of **P1** and **P2** are measured to be 11.6 kDa (polydispersity index (PDI) = 1.8) and 20.6 kDa (PDI = 2.0), respectively (Figures S4 and S5). The obtained polymers show good solubility in common organic solvents (e.g., chloroform, chlorobenzene, and *o*-dichlorobenzene). The thermal properties of the polymers were analyzed by thermogravimetric analysis (TGA) and differential scanning calorimetry (DSC) measurements. **P1** and **P2** showed the decomposition temperatures (*T*_d_) with 5% weight loss at 361.5 °C and 342.5 °C, respectively (Figure S6), indicating that both of **P1** and **P2** exhibited good thermal stability, and **P1** with polar side chain showed better thermal stability than **P2**. The difference in the decomposition temperatures was likely due to the different side chains on the benzothiodazole units, where the polar side chains might have stronger bond energy and form stronger van der Waals forces, as it is reported that the length of a CH_2_–O bond (1.42 Å) is slightly shorter than that of the CH_2_–CH_2_ bond (1.54 Å) [41]. No melting transitions were observed for both polymers in the differential scanning calorimentry (DSC) measurements in the temperature range of 25–300 °C, suggesting that **P1** and **P2** are both amorphous polymers (Figure S7).

**Figure 1:**
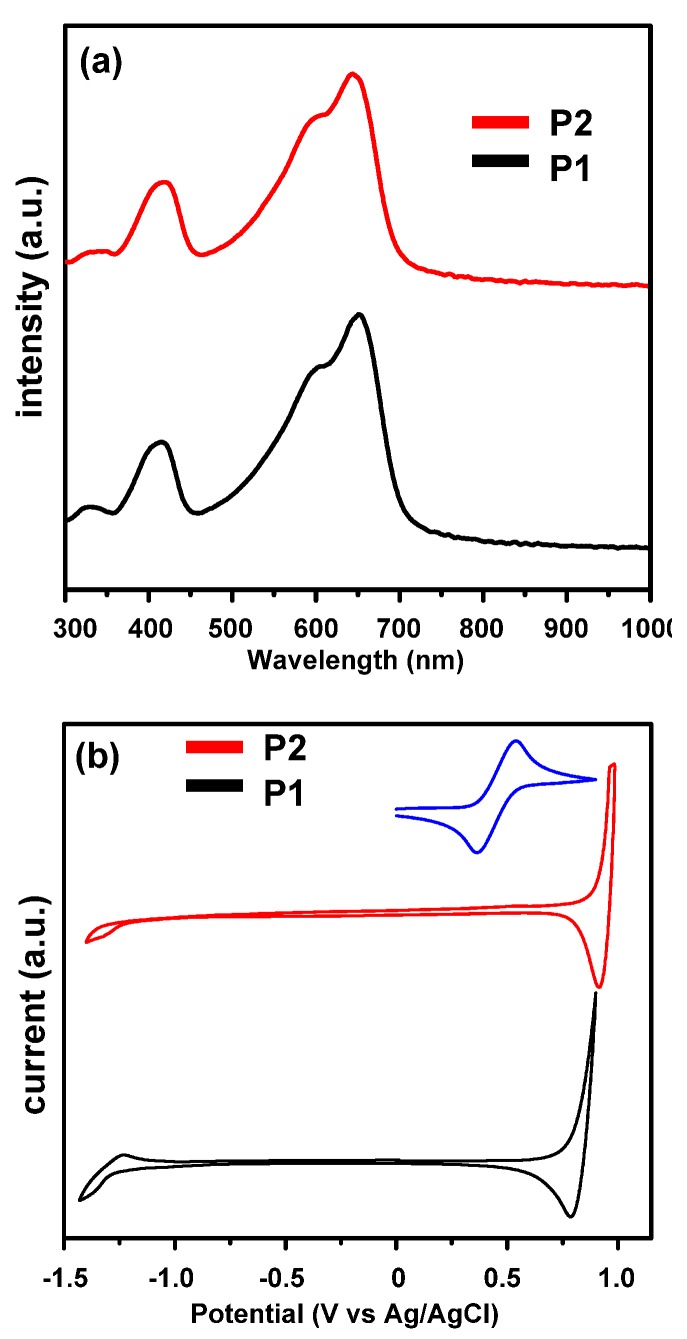
(**a**) Normalized UV-Vis absorption spectra of polymer films and (**b**) cyclic voltammograms (CV) of **P1** and **P2**.

Figure 1a shows the normalized UV-vis absorption spectra of **P1** and **P2** in film state, and the resulting optical properties are summarized in Table 1. In the film state, two polymers exhibited identical absorption spectra with a sharp shoulder at around 670 nm, which is attributed to the localized π–π* transitions due to the planar backbone of indacenodithiophene (IDT). The onsets of the absorption spectra for both **P1** and **P2** are 724 nm and 720 nm, respectively, indicating both of them have similar optical bandgaps (**P1**: 1.71 eV; **P2**: 1.72 eV, respectively, Table 1).

The similar absorption spectra suggest that the electronic structures of the two polymers remain constant when the side-chains change. By cyclic voltammetry (CV) measurements (Figure 1b), the HOMO/LUMO frontier orbital energy levels were determined to be −5.67 eV/−3.39 eV and −5.75 eV/−4.11 eV for **P1** and **P2**, respectively (Table 1). The lower oxidation potential of **P1** compared to that of **P2** should be attributed to the easy oxidation of the ethylene glycol ether side chain. The electronic bandgaps of **P1** and **P2** are determined to be 1.74 eV and 1.64 eV, respectively, confirming the aforementioned optical properties of the polymers.

### 3.2. Photophysical Properties of the CPs/SWCNT Composite Films

FT-IR spectra of **P1**/SWCNTs and **P2**/SWCNTs were recorded and are shown in Figure S10. In the case of **P1**/SWCNTs composites, the main absorption peaks of the polymers are described as follows: the absorption band of 560 cm^−1^ should be ascribed to the C-S bond of thiophene, the peak around 830 cm^−1^ is caused by the C–O–C bonds in the benzothiadiazole unit, the peak of 1255 cm^−1^ arises from the stretching vibration of unsaturated C–H bonds and the peak of 1629 cm^−1^ is from the C=N stretching vibration. **P2**/SWCNTs composite films exhibited similar absorption peaks as those of **P1**/SWCNTs composites, indicating that the electronic structures of polymers are not altered after adding different ratios of SWCNTs, namely, the polymers are quite stable in the composite films.

**Figure 2:**
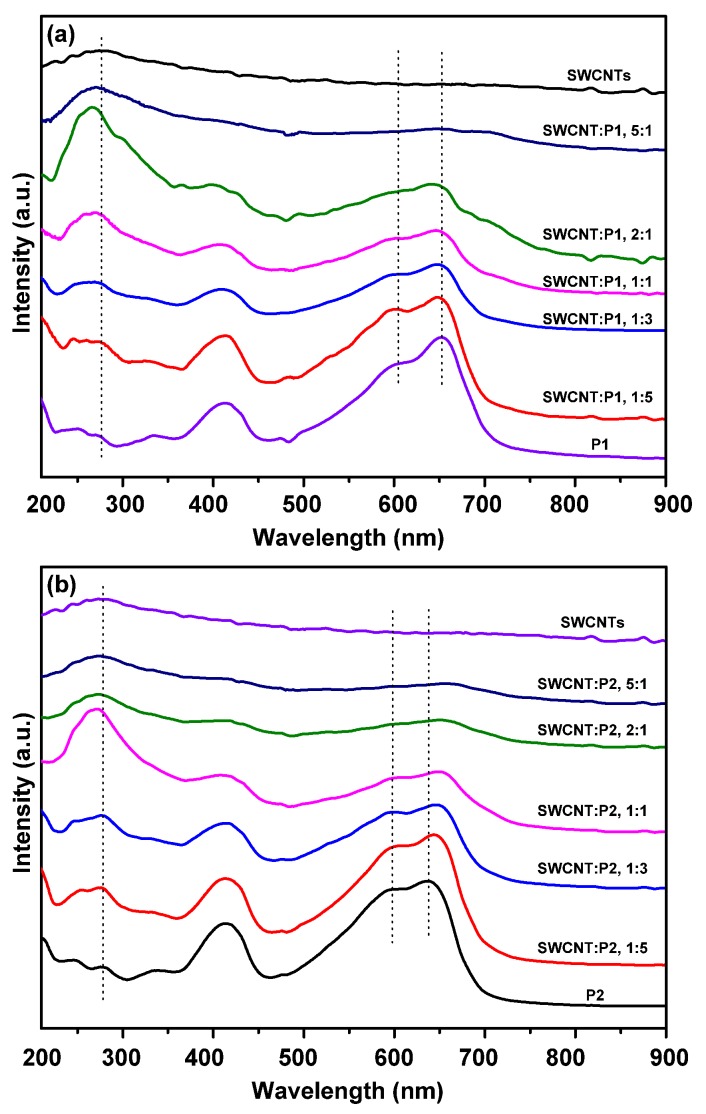
UV-Vis absorption spectra of (**a**) **P1**/SWCNTs and (**b**) **P2**/SWCNTs composite films with varying the amounts of SWCNTs.

The UV-Vis absorption spectra were recorded to study the electronic band structures and interfacial interactions of **P1**/SWCNTs and **P2**/SWCNTs composites. The peak at around 270 nm should be assigned to SWCNTs, and its intensity was enhanced upon increasing the amounts of the SWCNTs (Figure 2a,b), accompanied by a shifting of the spectra by a few nanometers. The peaks at around 600 nm and 650 nm for **P1**/SWCNT composites shifted to small wavelengths as the amounts of SWCNTs increased, however, we noticed a small red shift of the peaks at around 600 nm and 650 nm to longer wavelengths for **P2**/SWCNTs, indicating that different packing styles existed in the **P1**/SWCNT and **P2**/SWCNT composites, and we speculate that the different packing features should be caused by the more intensified interfacial interactions of **P1**/SWCNTs than that of **P2**/SWCNTs, although the detailed packing features of the composites are still unclear at the current stage.

### 3.3. Surface Morphologies of the CPs/SWCNT Composite Films

**Figure 3:**
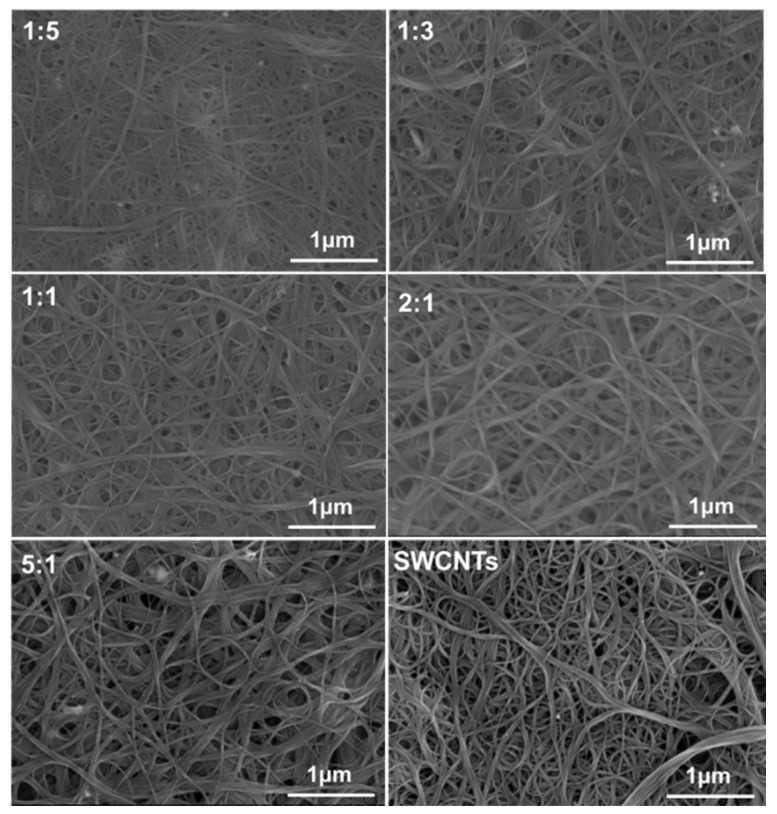
Scanning electron microscope (SEM) images of **P1**/SWCNTs composite films with different amounts of SWCNTs.

In order to investigate the effects of surface morphologies on the properties of the composites, the surface morphologies of CPs/SWCNT were characterized by field scanning electron microscopy (FE-SEM) as shown in Figure 3 and Figure S9. It is clear that the SWCNTs bundles are distributed homogeneously on the composite films. Fibrillar structures are clearly observed from the images shown in Figure 3, which could be attributed to the successfully wrapping of polymers onto the SWCNTs surfaces through strong interfacial interactions between them. With increasing amounts of SWCNTs, the diameters of the bundled fibers becomes smaller, and the diameter of the pristine SWCNTs is the smallest one. Compared to **P1**/SWCNT composites, the surface morphology of **P2**/SWCNT as shown in Figure S9 exhibited small aggregates due to the relatively poor interactions between them. These results indicated that the existence of a polar side chain can be beneficial to form homogeneous composite films through the enhanced interfacial interactions between the polymer and SWCNTs.

### 3.4. Raman Spectroscopies of P1/SWCNTs and P2/SWCNTs Composites

**Figure 4:**
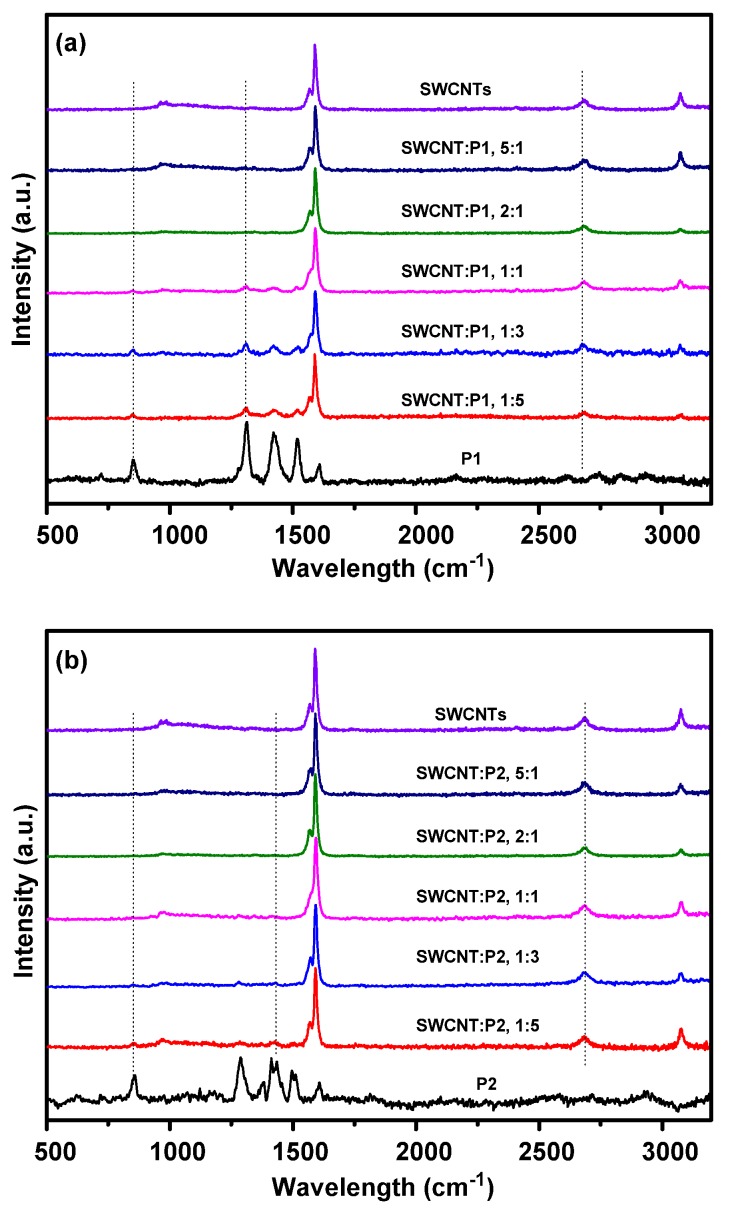
Raman spectra of (**a**) **P1**/SWCNTs composites and (**b**) **P2**/SWCNTs composites films with different amounts of SWCNTs.

The Raman spectra of pristine polymers, SWCNTs, and the CPs/SWCNT composite films with different mass ratios of polymers to SWCNTs are presented in Figure 4 and Figure S8. The spectra of the pure SWCNTs showed characteristic peaks at 1562 and 1593 cm^−1^, respectively, where the peak at 1593 cm^−1^ could be assigned to the G band of SWCNTs that originates from the splitting of the *E*_2g_ stretching mode of graphite. Some important characteristic peaks were observed for the pristine **P1** and **P2** film. The peaks at around 1519 and 1610 cm^−1^ for **P1** and 1510 and 1608 cm^−1^ for **P2** should be attributed to the benzene ring stretching vibrations of the IDT and BT units. The peaks at around 1312 and 1421 cm^−1^ correspond to the C=C symmetrical stretching mode of the thiophene segments for **P1** and **P2**, respectively, and the peaks at 850 and 857 cm^−1^ could be attributed to the C–C stretching vibrations of **P1** and **P2**. In the case of composites films, the intensity of the characteristic peaks of the pristine **P1** and **P2** gradually decreased and eventually disappeared when the amount of SWCNTs was increased. We noticed that the G band peak of SWCNT showed shifts of approximately 2–5 cm^−1^ in the composite films of **P1**/SWCNTs, which is hardly observed in **P2**/SWCNTs composites, resulting from the strong π–π interfacial interactions between the polymers and SWCNTs (Figure S8).

**Figure 5:**
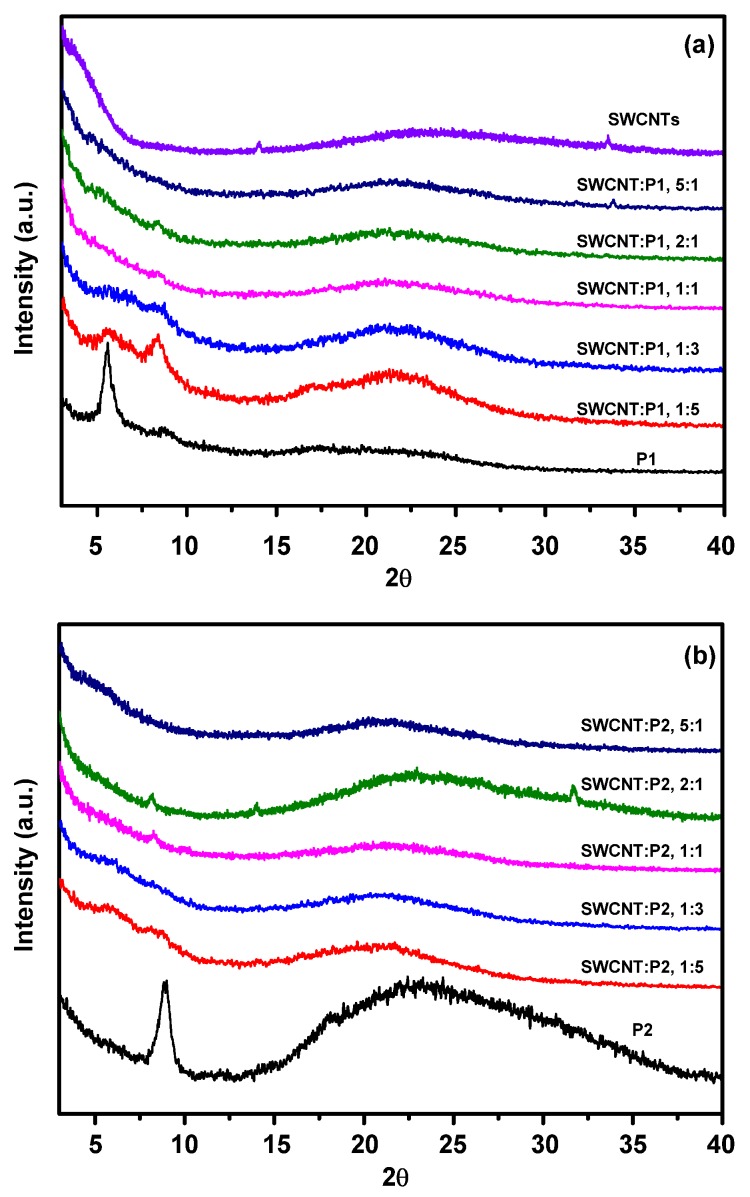
Grazing incidence X-ray diffraction (GI-XRD) spectra of the (**a**) pristine **P1** and its SWCNT composites and (**b**) pristine **P2** and its SWCNT composites.

In order to further investigate the interfacial interactions between polymers and the SWCNTs, grazing-incident X-ray diffraction (GI-XRD) was performed on the pristine **P1**, **P2** and their SWCNTs composites films as shown in Figure 5. There are no obvious π–π stacking peaks for pristine **P1** and **P2** films, confirming the amorphous features of both polymers as confirmed by DSC. **P1** showed a halo peak between 15° to 30°, which is narrower than the halo peak of **P2** (between 15° to 40°), and in the meantime, there is a sharp peak at around 5.46° for **P1** and around 8.96° for **P2**, indicating that the interpolymer chain distance of **P1** is larger than that of **P2**. The larger interpolymer distance of **P1** indicates that **P1** tends to form looser packing than that of **P2**, which is beneficial to form homogenous surface morphologies with SWCNTs. In the XRD patterns of the **P1**/SWCNTs composite films, the sharp peak at around 5.46° is significantly decreased as the amount of SWCNTs is increased, indicating that the polymers tend to bind with SWCNTs. The typical diffraction peak of pure SWCNT at 2θ = 26.5° moved to a slightly lower angle, and the intensity of the diffraction peak also decreased, which further proved the presence of strong π–π interfacial interactions between **P1** and SWCNTs, however, no obvious shifting of the peak was observed for **P2**/SWCNTs composite films.

### 3.5. Thermoelectric Properties of P1/SWCNT and P2/SWCNT Composites

**Figure 6:**
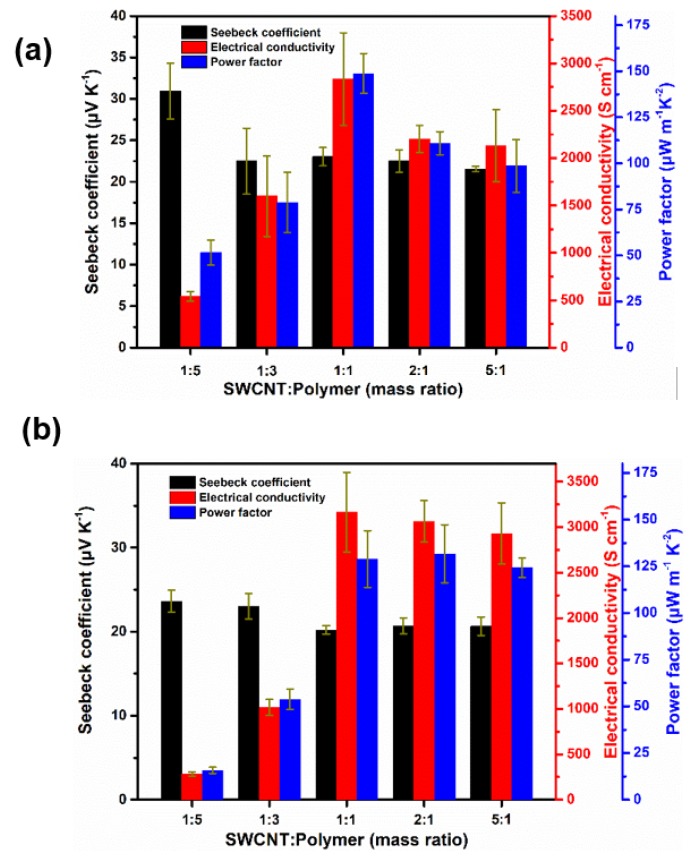
Electrical conductivities, Seebeck coefficients, and power factor values of **P1**/SWCNT (**a**) and **P2**/SWCNT composite films (**b**).

The electrical conductivities and Seebeck coefficients are presented in Figure 6. The introduction of SWCNTs significantly improved the electrical conductivity of the composites. Both the **P1**/SWCNT and **P2**/SWCNT composites with 50% SWCNTs exhibited the highest values of electrical conductivities, 3392.67 S cm^−1^ and 3283.09 S cm^−1^, respectively. These enhanced electrical conductivities could be generated from the formed homogeneous network structure of the composites films as shown in the SEM images (Figure 3). Further increasing the amount of SWCNTs was detrimental to the electrical conductivities of both of the **P1**/SWCNT and **P2**/SWCNT composites, probably due to the aggregation of SWCNTs that breaks the network structure of composites films. As shown in Figure 6, the maximum power factor value of 161.34 μW m^−1^ K^−2^ for **P1**/SWCNT (1:1, weight ratio) is higher than that of **P2**/SWCNT (139.06 μW m^−1^ K^−2^) for the same ratio of the polymer and SWCNTs, due to the much larger Seebeck coefficient value of **P1**/SWCNTs composites. The maximum power factor value (144.77 μW m^−1^ K^−2^) for **P2**/SWCNT (2:1, weight ratio) is still lower than the maximum value of **P1**/SWCNTs. These results indicated that the incorporation of polar side chains to the indacenodithiophene-benzothiazole copolymers could improve the electrical conductivities as well as maintain the large Seebeck coefficient value of the polymers/SWCNTs composites, leading to enhanced thermoelectric performance.

## 4. Conclusions

Two indacenodithiophene and benzothiazole alternating copolymers with triethylene glycol ether side chains (**P1**) and aliphatic side chains (**P2**) were synthesized and their corresponding composites with SWCNTs were prepared. Compared with the properties of **P2** with an aliphatic side chain, **P1** exhibited similar photophysical features, electronic properties and bandgaps. After forming composites with SWCNTs, **P1** with a polar side chain possesses much stronger interactions with SWCNTs than those of **P2**, which has been evidenced by their UV-Vis, XRD, and Raman spectra characterizations. **P1** with 50 wt% SWCNTs exhibited a PF value of 161.34 μW m^−1^ K^−2^ which is higher than the value of **P2**(139.06 μW m^−1^ K^−2^) for the same amount of added SWCNTs. These results confirmed that the incorporation of polar side chains into the conjugated polymer backbone is an effective strategy to build thermoelectric conjugated polymer/SWCNT composites with high performance.

## Figures and Tables

**Table 1:** Summary of the Photophysical, Electrochemical, and Thermal Properties of **P1** and **P2**.

Polym.	*M*_n_^a^ (kg mol^−1^)	PDI	*E*^ox^ (eV)	*E*^red^ (eV)	*E*_HOMO_^b^ (eV)	*E*_LUMO_ (eV)	*E*ec (eV)	*λ*^onset^ (nm)	*E*^op^tg (eV)
**P1**	11.6	1.8	0.73	−1.01	−5.67	−3.39	1.74	724	1.71
**P2**	20.6	2.0	0.81	−0.83	−5.75	−4.11	1.64	720	1.72

^a^ Number-average molecular weight (*M*_n_) determined by SEC. ^b^ The HOMO energy levels of the polymers can be calculated from the *E_onset_* for oxidation (*E_ox,onset,polymer_*) using the equation: *E_HOMO_* = −*E*(*E_ox,onset,polymer_* − *E_1/2,ferrocene_*) + (−5.39 ev). The LUMO energy levels are obtained using the following equation: *E_LUMO_* = −*E*(*E_re,onset,polymer_* − *E_1/2,ferrocene_*) + (−5.39 ev). The band gap *E_g_* is calculated from the equalality: *E_g_* = *E_LUMO_* − *E_HOMO_*.

## Supplementary Materials

The following are available online at https://www.mdpi.com/2073-4360/12/4/848/s1, Figure S1: ^1^H NMR spectrum of **M1**, Figure S2: ^1^H NMR spectrum of **P1**, Figure S3: ^1^H NMR spectrum of **P2**, Figure S4: GPC curve of **P1**, Figure S5: GPC curve of **P2**, Figure S6: TGA curves of **P1** and **P2**, Figure S7: DSC curves of **P1** and **P2**, Figure S8: The G-band magnification of **P1** films, SWCNTs, the **P1**/SWCNT composites films (a) and **P2** films, SWCNTs, the **P2**/SWCNT composites films (b), Figure S9: SEM images of **P2**/ SWCNT composite films with different mass ratios of SWCNTs, Figure S10: FT-IR spectra of **P1**/SWCNT composite films with different mass ratios of SWCNTs (a) and **P2**/SWCNT composite films with different mass ratios of SWCNTs (b).
