# Supplementary material for: Enhanced Thermoelectric Performance of Indacenodithiophene-Benzothiadiazole Copolymer Containing Polar Side Chains and Single Wall Carbon Nanotubes Composites
Source: Polymers (Basel). 2020 Apr 7;12(4):848. doi: 10.3390/polym12040848 (PMC7240368; doi:10.3390/polym12040848)
Supplement: Supplementary file 1 [file polymers-12-00848-s001.pdf]

Enhanced Thermoelectric Performance of Indacenodithiophene-Benzothiadiazole Copolymer Containing Polar Side Chains and Single Wall Carbon Nanotubes Composites

Zhongming Chen, Tongchao Liu, Chengjun Pan and Guiping Tan

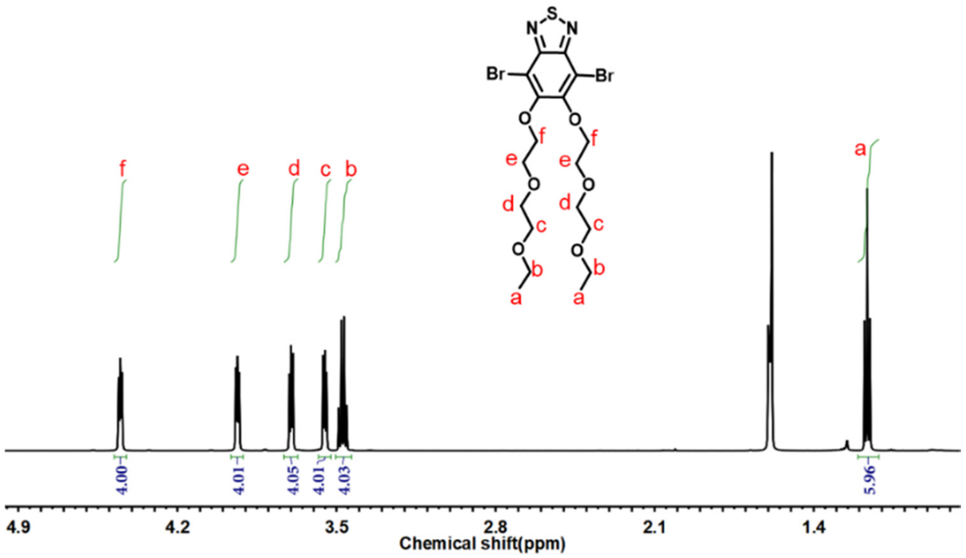

Figure S1. ^{1}H NMR spectrum of **M1**.

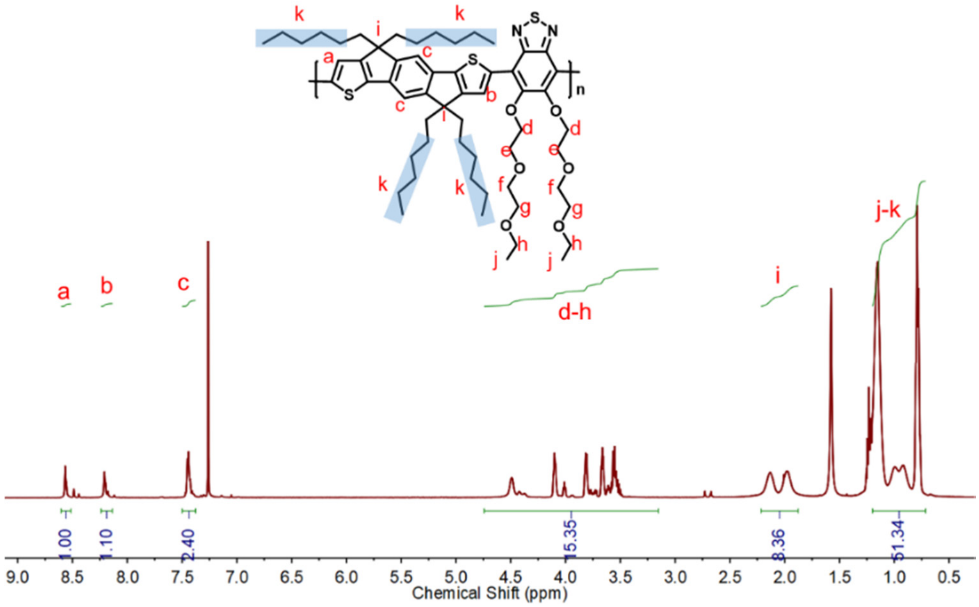

Figure S2. ^{1}H NMR spectrum of **P1**.

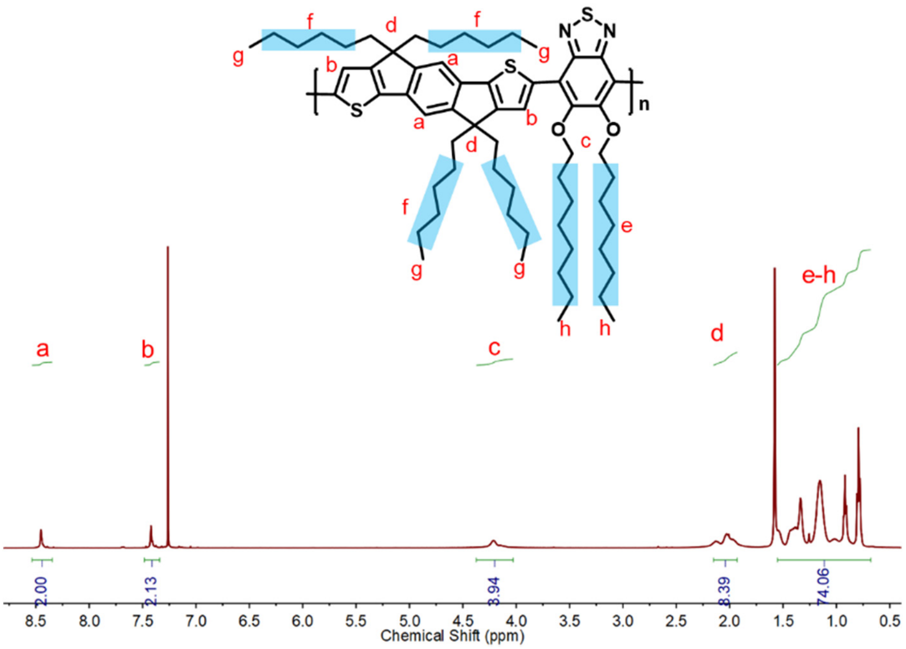

Figure S3. ^{1}H NMR spectrum of **P2**.

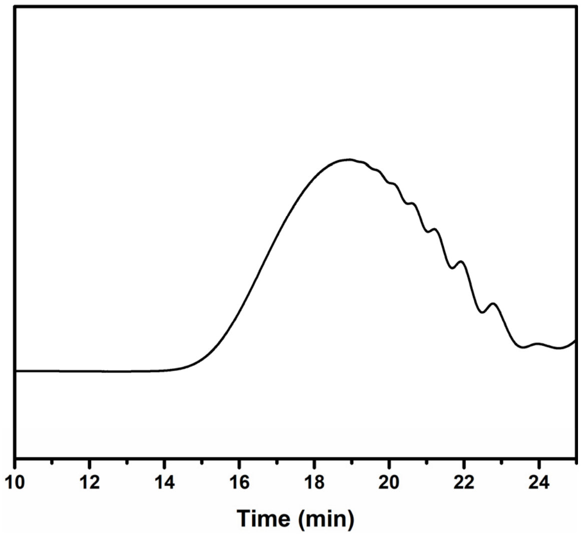

Figure S4. GPC curve of P1.

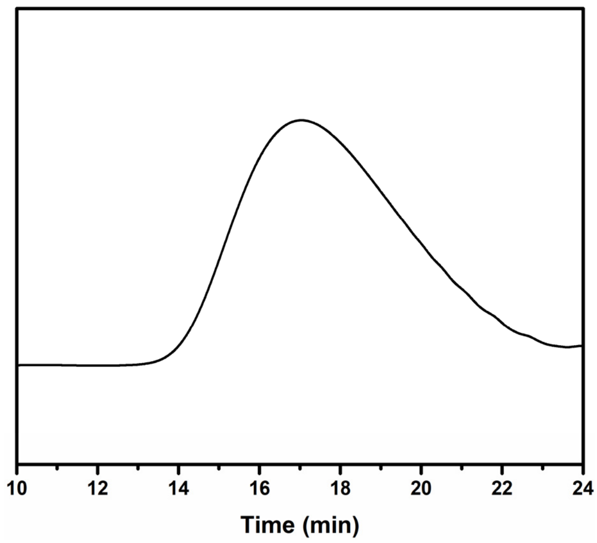

Figure S5. GPC curve of P2.

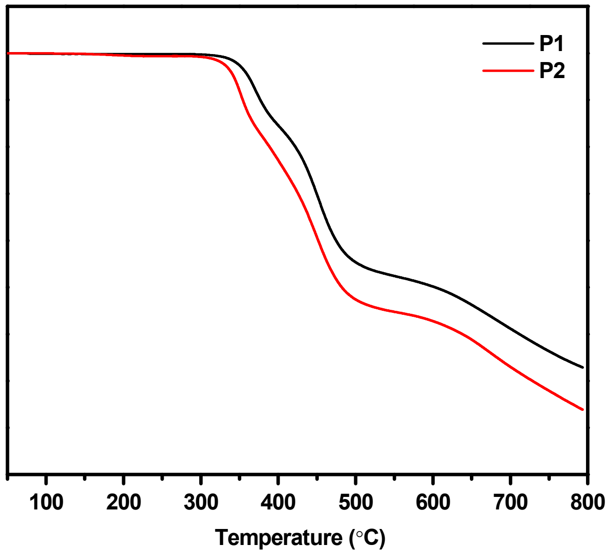

Figure S6. TGA curves of **P1** and **P2**.

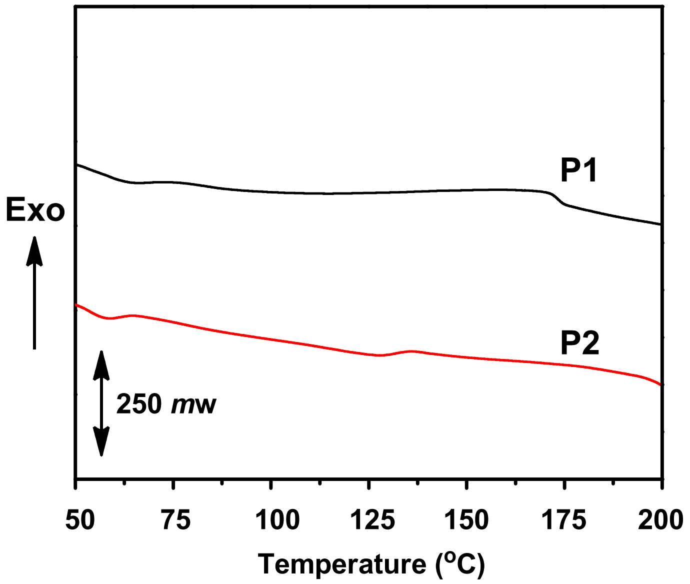

Figure S7. DSC curves of **P1** and **P2**.

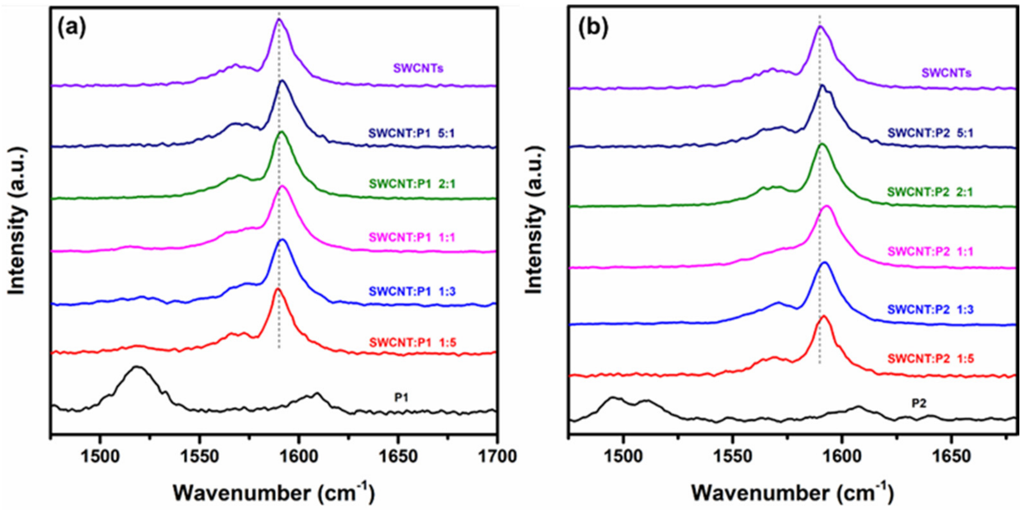

Figure S8. The G-band magnification of **P1** films, SWCNTs, the **P1**/SWCNT composites films (**a**) and **P2** films, SWCNTs, the **P2**/SWCNT composites films (**b**).

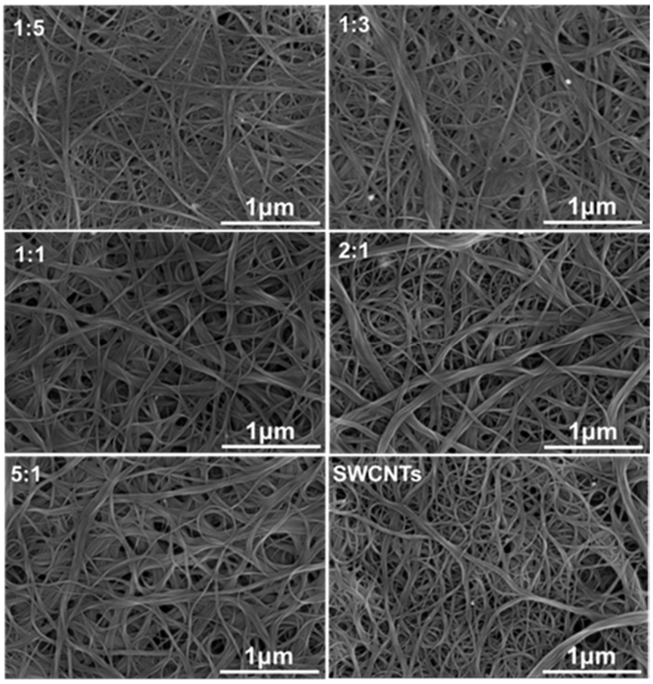

Figure S9. SEM images of **P2**/ SWCNT composite films with different mass ratios of SWCNTs.

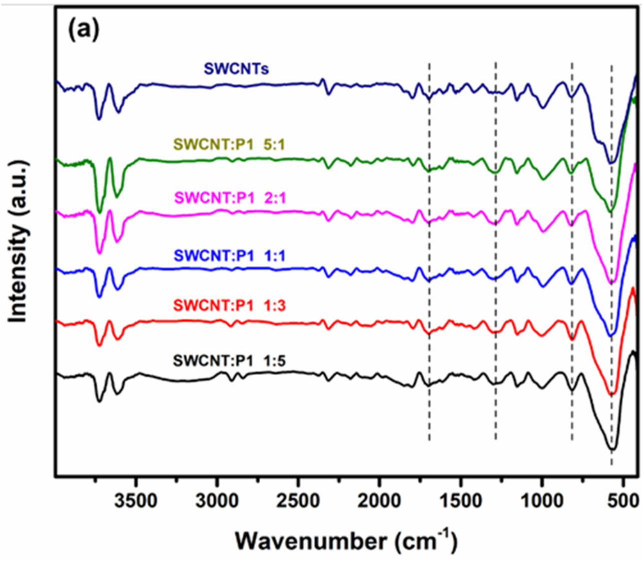

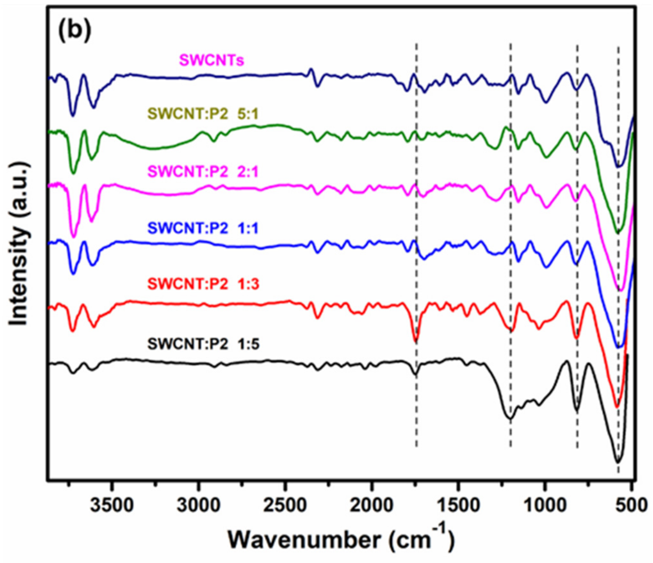

Figure S10. FT-IR spectra of **P1**/SWCNT composite films with different mass ratios of SWCNTs (**a**) and **P2**/SWCNT composite films with different mass ratios of SWCNTs (**b**).
